# Long non-coding RNA NORAD protects against cerebral ischemia/reperfusion injury induced brain damage, cell apoptosis, oxidative stress and inflammation by regulating miR-30a-5p/YWHAG

**DOI:** 10.1080/21655979.2021.1995115

**Published:** 2021-11-22

**Authors:** Xinyu Zhou, Zhonglong Wang, Bingchao Xu, Niu Ji, Pin Meng, Lei Gu, Ying Li

**Affiliations:** a Department of Neurology, The Affiliated Lianyungang Hospital of Xuzhou Medical University, the First People’s Hospital of Lianyungang, Lianyungang, Jiangsu Province, China; b Department of Neurology, Jining Psychiatric Hospital, Jining, Shandong Province, China; c Rehabilitation Center, Beijing Xiaotangshan Hospital, Beijing, China

**Keywords:** LncRNA NORAD, miR-30a-5p, YWHAG, OGD/R injury, cerebral ischemia/reperfusion injury

## Abstract

LncRNAs are identified as critical regulators in cerebral ischemia/reperfusion injury (CIRI). In this current work, SH-SY5Y cells suffered from oxygen-glucose deprivation/reperfusion (OGD/R) were applied to analyze the biological role of lncRNA NORAD and underlying molecular mechanism in CIRI in vitro. Levels of lncRNA NORAD, miR-30a-5p and YWHAG were measured using RT-qPCR. Bioinformatics analysis predicted the binding sites of lncRNA NORAD to miR-30a-5p and miR-30a-5p to YWHAG. Luciferase reporter assay verified the binding relationships among lncRNA NORAD, miR-30a-5p and YWHAG. Additionally, cell viability was determined using CCK-8 assay, and cell apoptosis was assessed using TUNEL staining and western blot analysis. Moreover, the levels of ROS, MDA, LDH and SOD as well as IL-1β, TNF-α, and IL-6 were assessed via application of the corresponding assay kits. Decreased cell viability and temporarily increased lncRNA NORAD level were observed in SH-SY5Y cells after OGD/R. It was demonstrated that lncRNA NORAD regulated YWHAG expression by sponging miR-30a-5p. Upregulation of lncRNA NORAD contributed to the enhancement of cell viability, the inhibition of cell apoptosis as well as the alleviation of oxidative stress and inflammation in OGD/R-injured SH-SY5Y cells, which were reversed upon elevation of miR-30a-5p. In contrast, downregulation of lncRNA NORAD reduced cell viability, promoted cell apoptosis as well as aggravated oxidative stress and inflammation under OGD/R challenge, and the functions of lncRNA NORAD knockdown in OGD/R injury were abolished by upregulation of YWHAG. Taken together, lncRNA NORAD exerted protective effects against OGD/R-induced neural injury by sponging miR-30a-5p to upregulate YWHAG expression.

## Introduction

Stroke seriously threatens public health globally. Ischemic stroke, also known as cerebral ischemia, is the most common form of stroke, featuring high morbidity, disability and mortality rates [1]. After cerebral ischemia, brain damage emerges due to the shortage of oxygen and nutrients from the blood [2]. Currently, restoring blood flow is the most effective therapeutics for ischemic stroke, which in turn evokes nerve cell damage and death [3]. This phenomenon is defined as cerebral ischemia-reperfusion injury (CIRI) [4]. CIRI involves a variety of pathophysiological mechanisms. It is believed that the recovery of the circulation induces the accumulation of metabolic toxins, oxidative stress, inflammation and apoptosis, thereby aggravating brain damage [5–7]. Thus, developing effective therapeutic targets or measures against nerve damages caused by CIRI is a task of top priority.

Long non-coding RNAs (lncRNAs) are a group of non-coding RNAs that consist of more than 200 nucleotides [8]. It has been reported that a large amount of lncRNAs are abnormally expressed during the development of nervous system diseases [9,10]. Song et al. [11] has proved that upregulation of lncRNA NORAD could protect Parkinson’s disease model cells against cellular apoptosis induced by MPP+. However, the biological role and molecular mechanisms underlying lncRNA NORAD in the progression of CIRI are yet to be fully elucidated.

Studies have reported that a variety of lncRNAs have a sponge adsorption effect on microRNAs (miRNAs) and thus participate in the process of CIRI [12,13]. The interaction between lncRNA NORAD and miR-30a-5p was predicted on the online tool starBase (http://starbase.sysu.edu.cn/index.php). Besides, miR-30a has been proved to be deregulated in patients suffering from ischemic stroke [14]. Furthermore, Wang et al. [15] indicated that the inhibition of miR-30a-5p could prevent cerebral ischemia-induced BBB damage, reduce infarct volume and ameliorate neurological deficits.

What is more, 14-3-3γ (YWHAG) was predicted as a target gene of miR-30a-5p (starBase). 14-3-3 proteins are composed of cellular proteins of high conservation and are broadly introduced in different eukaryotes [16]. 14-3-3 proteins have been divided into seven isoforms, among which 14-3-3γ isoform is also called tyrosine 3-monooxygenase/tryptophan 5-monooxygenase activation protein gamma (YWHAG) [17]. Increasing evidence verifies that 14-3-3 proteins bring about tremendous influence on various nervous system diseases. Literature has reported that recurrent distal 7q11.23 including YWHAG is deleted in patients with neurological dysfunction [18]. Besides, YWHAG knockdown is sufficient to induce cell death in normal cultured neurons and exacerbate OGD-induced neuronal death [19]. Moreover, selectively upregulated YWHAG could be translocated into the nuclei of neurons after CIRI to protect neurons against I/R injury by regulating NF-κB signaling [20].

To conclude, this present work was carried out to identify the biological functions of lncRNA NORAD/miR-30a-5p/YWHAG axis in CIRI and to investigate the molecular mechanisms underlying lncRNA NORAD/miR-30a-5p/YWHAG in the development of CIRI.

## Materials and methods

### Cell culture

The American Type Culture Collection (ATCC, VA, USA) was the provider of human neuroblastoma SH-SY5Y cells. In a word, Dulbecco’s Modified Eagle Medium (DMEM; HyClone, UT, USA) which contains 10% fetal bovine serum (FBS; Gibco, NY, USA) and 1% penicillin/streptomycin solution (Gibco, NY, USA) was applied to culture SH-SY5Y cells at 37°C in a humidified atmosphere containing 5% CO_2_.

### Cell transfection

The NORAD shRNA (sh-NORAD), NORAD overexpression vector (pcDNA3.1-NORAD), miR-30a-5p mimic, YWHAG overexpression vector (Ov-YWHAG) and their corresponding negative control were obtained from GenePharma (Shanghai, China). When cells reached 80% confluence, transfection was conducted by Lipofectamine 2000 (Invitrogen, CA, USA) in strict line with the manufacturer’s protocol.

### Oxygen-glucose deprivation/reperfusion (OGD/R) model

In order to mimic I/R procedure *in vitro*, OGD/R injuries were carried out. In detail, SH-SY5Y cells were cultured for 2, 4, 6, 8, 10, 12 h in glucose-free DMEM medium under hypoxic conditions (1% O_2_, 5% CO_2_, 94% N_2_). Subsequently, the cells were returned to normal DMEM medium and subjected to reoxygenation (21% O_2_, 5% CO_2_, 74% N_2_) continuously.

### Cell counting kit-8 (CCK-8) assay

Viability of SH-SY5Y cells was measured via application of CCK-8 assay. After OGD/R and designed transfection, 10 μL of CCK-8 reagent was added into each well for an additional 3 h incubation. Under the circumstance of λ = 450 nm, the absorbance of each well was detected with the application of microplate reader (Bio-Rad, CA, USA).

### Real-time quantitative polymerase chain reaction (RT-qPCR)

RNA isolation from cultured SH-SY5Y cells was performed with TRIzol reagent in line with the instructions put forward by the manufacturer. Equal amount of RNA was reversely transcribed into cDNA using a reverse transcription kit (Takara, Tokyo, Japan). Quantification of gene expression by RT-qPCR was performed on the ABI PRISM 7000 Sequence Detection System (ABI/Perkin Elmer, CA, USA). The PCR conditions were as follows: denaturation at 95°C for 20 s, 30 cycles of denaturation at 95°C for 30 s, annealing at 55°C for 30 s and extension at 72°C for 30 s. The primer sequences were listed as follows: NORAD: forward 5ʹ-TGATAGGATACATCTTGGACATGGA-3ʹ; reverse 5ʹ-AACCTAATGAACAAGTCCTGACATACA-3ʹ; miR-30a-5p: forward 5ʹ-GGGCCTGTAAACATCCTCG-3ʹ; reverse 5ʹ-GAATACCTCGGACCCTGC-3ʹ; YWHAG: forward 5ʹ-GCCGTATGTCAGGATGT-3ʹ; reverse 5ʹ-GCCAGGTAGCGGTAAT-3ʹ; GAPDH: forward 5ʹ-CTGCACCACCAACTGCTTAG-3ʹ; reverse 5ʹ-AGGTCCACCACTGACACGTT-3ʹ; U6: forward 5ʹ- GCTTCGGCAGCACATATACT −3ʹ; reverse 5ʹ- GTGCAGGGTCCGAGGTATTC −3ʹ. GAPDH served as the endogenous control for NORAD and YWHAG and U6 served as the endogenous control for miR-30a-5p. The gene expression levels were calculated using the 2^−∆∆Ct^ method [21].

### TUNEL assay

Apoptosis of SH-SY5Y cells was measured via application of TUNEL staining. In short, SH-SY5Y cells were fixed with 4% paraformaldehyde for 30 min. Then, cells were permeabilized in 0.1% Triton X-100 solution for 2 min on ice. Next, cells were incubated with TUNEL reaction compound (Roche, Basel, Switzerland) for 1 h at 37°C. After staining with 4, 6-diamidino-2-phenylindole (DAPI; Solarbio, Beijing, China) for 10 min in the dark, a fluorescence microscope (Olympus, Tokyo, Japan) was applied to observe and photograph the TUNEL-positive cells.

### Western blot assay

Protein extraction from SH-SY5Y cells was performed using radioimmunoprecipitation assay lysing solution and protein concentration was examined by Bicinchoninic acid (BCA; Beyotime, Shanghai, China) method. After electrophoresis on SDS-PAGE, proteins were transferred to PVDF membranes. Membranes were incubated overnight at 4°C with corresponding primary antibodies against Bcl-2 (Abcam, ab196495, 1:2000), Bax (Abcam, ab53154, 1:1000), Cleaved caspase-3 (Abcam, ab49822, 1:500), Caspase-3 (Abcam, ab13847, 1:500) and GAPDH (Abcam, ab181602, 1:10,000) and incubated with anti-rabbit or anti-mouse IgG horseradish peroxidase-linked antibodies at room temperature for 1 h. Signals were developed and detected via application of electrochemiluminescence (ECL; Beyotime, Shanghai, China) method. Relative protein levels were quantified by densitometry using GAPDH as the endogenous control.

### Reactive oxygen species (ROS) measurement

The content of ROS in cell suspension was determined by ROS Assay Kit in strict line with the manufacturer’s protocol. Cells were stained with 5 μmol/L 2ʹ, 7ʹ-dichlorofluorescin diacetate (DCFH-DA; Beyotime, Shanghai, China) for 30 min at 37°C. After washing and re-suspending in PBS, the content of ROS was assessed on flow cytometry (BD Biosciences, CA, USA).

### Malondialdehyde (MDA), lactate dehydrogenase (LDH) and superoxide dismutase (SOD) measurement

The levels of MDA, LDH as well as SOD in cell suspension were separately assessed by MDA (A003-1) Assay Kit, LDH (A020-2) Assay Kit and SOD (A001-3) Assay Kit (Jiancheng Biotechnology Research Institute, Nanjing, China) in strict line with the manufacturer’s protocol.

### Enzyme-linked immunosorbent assay (ELISA)

In order to determine the release of inflammatory cytokines, ELISA Kits (Beyotime, Shanghai, China) were employed to measure the levels of tumor necrosis factor-α (TNF-α), Interleukin (IL)-1β and IL-6. Under the circumstance of λ = 450 nm, the optical density was recorded on a microplate reader (Bio-Rad, CA, USA).

### Luciferase reporter assay

The binding sites of lncRNA-NORAD to miR-30a-5p and miR-30a-5p to YWHAG were analyzed on bioinformatics website. NORAD-WT and NORAD-MUT 3ʹ-UTRs were constructed and cloned into the luciferase vectors. Co-transfection of miR-30a-5p mimic or miR-NC and the luciferase vectors into SH-SY5Y cells was carried out using Lipofectamine 2000 (Invitrogen, USA). Dual Luciferase Reporter Assay System (Promega, WI, USA) was employed to measure the luciferase activity. The binding relationship between miR-30a-5p and YWHAG was verified following the method described above.

### Statistical analysis

All experimental data were displayed as mean values ± standard deviation (SD) of three replicates. One-way analysis of variance (ANOVA) followed by Tukey’s post hoc test was adopted to conduct the statistical comparisons among multiple groups. A *p*-value below 0.05 was recognized as statistical significance.

## Results

### Decreased cell viability and temporarily increased lncRNA NORAD level after OGD/R induction

**Figure 1.**
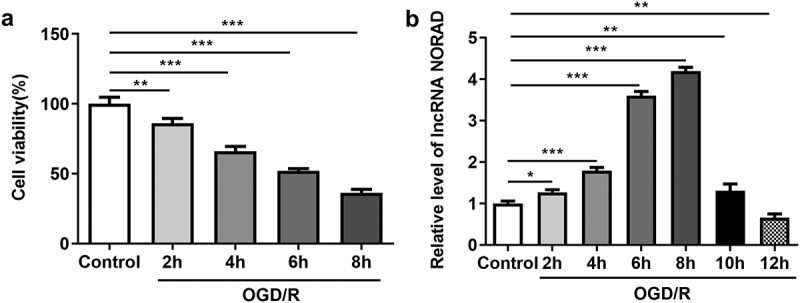
Decreased cell viability and temporarily increased lncRNA NORAD level after OGD/R induction. SH-SY5Y cells were cultured for 2, 4, 6, 8, 10, 12 h in glucose-free DMEM medium under hypoxic conditions (1% O_2_, 5% CO_2_, 94% N_2_). Subsequently, the cells were returned to normal DMEM medium and subjected to reoxygenation (21% O_2_, 5% CO_2_, 74% N_2_) continuously. (a) Cell viability was determined using CCK8 assay. (b) LncRNA NORAD level was detected by RT-qPCR. *P < 0.05, **P < 0.01, ***P < 0.001

To further elucidate the biological role of lncRNA NORAD in the cerebral ischemia/reperfusion injury, we measured the cell viability and lncRNA NORAD level in OGD/R-injured SH-SY5Y cells. Cell viabilities were time-dependently decreased following 2, 4, 6, 8 h OGD/R injury (Figure 1(a)). LncRNA NORAD expression temporarily increased following 2, 4, 6, 8 h OGD/R injury in a time-dependent manner while lncRNA NORAD expression notably decreased following 10, 12 h OGD/R injury (Figure 1(b)). These results indicated that temporary increase of lncRNA NORAD level in SH-SY5Y cells injured by OGD/R might act as a neuroprotective player in CIRI.

### Upregulation of lncRNA NORAD enhanced cell viability and suppressed cell apoptosis under OGD/R condition

**Figure 2.**
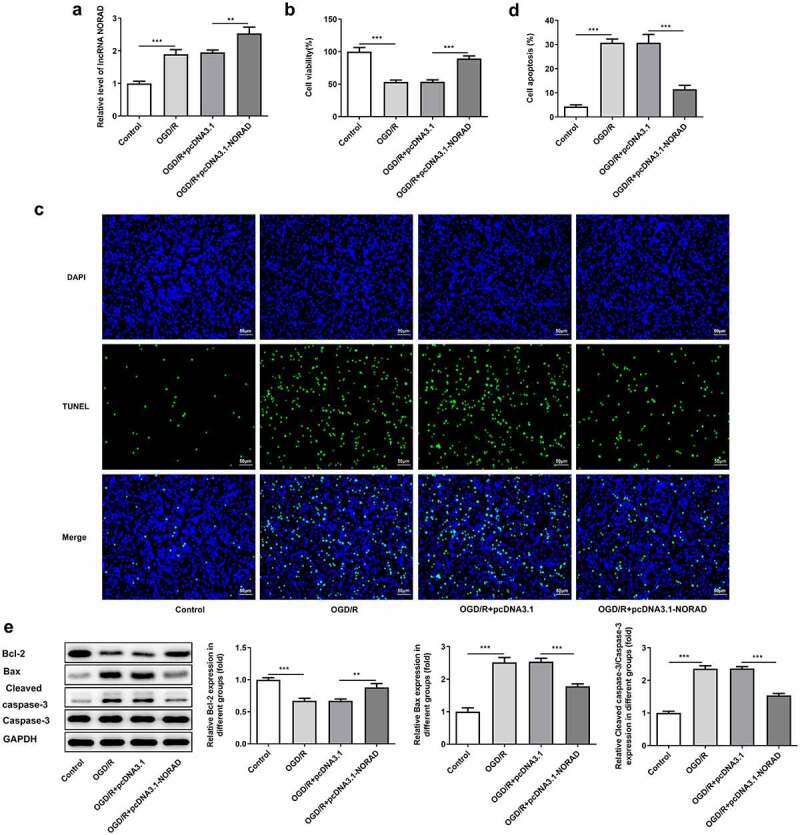
Upregulation of lncRNA NORAD enhanced cell viability and suppressed cell apoptosis under OGD/R condition. OGD/R-injured SH-SY5Y cells were transfected with pcDNA3.1-NC or pcDNA3.1-NORAD. (a) The overexpression efficiency was validated by RT-qPCR analysis. (b) Cell viability was determined using CCK-8 assay. (c, d) Cell apoptosis was assessed using TUNEL assay. (e) Expression levels of Bcl-2, Bax and Cleaved caspase-3 were detected using western blot assay. **P < 0.01, ***P < 0.001

In order to identify the biological function of lncRNA NORAD in CIRI, SH-SY5Y cells were transfected with pcDNA3.1-NORAD and the overexpression efficiency was validated by RT-qPCR analysis (Figure 2(a)). Viability of SH-SY5Y cells was obviously decreased upon 6 h OGD/R injury while upregulation of lncRNA NORAD enhanced cell viability, reversing OGD/R injury (Figure 2(b)). Cell apoptosis manifested as the number of TUNEL positive cells was extremely increased in OGD/R-injured SH-SY5Y cells, which could be mitigated by upregulating lncRNA NORAD (Figure 2(c, d)). Moreover, results of western blot analysis demonstrated that upregulation of lncRNA NORAD caused an increase in the expression of anti-apoptotic protein Bcl-2 as well as a decrease in the expressions of pro-apoptotic protein Bax and Cleaved caspase-3 (Figure 2(e)). Findings above collectively evidenced that lncRNA NORAD overexpression enhanced the viability and suppressed the apoptosis of OGD/R-injured SH-SY5Y cells.

### Downregulation of lncRNA NORAD reduced cell viability and promoted cell apoptosis under OGD/R condition

**Figure 3.**
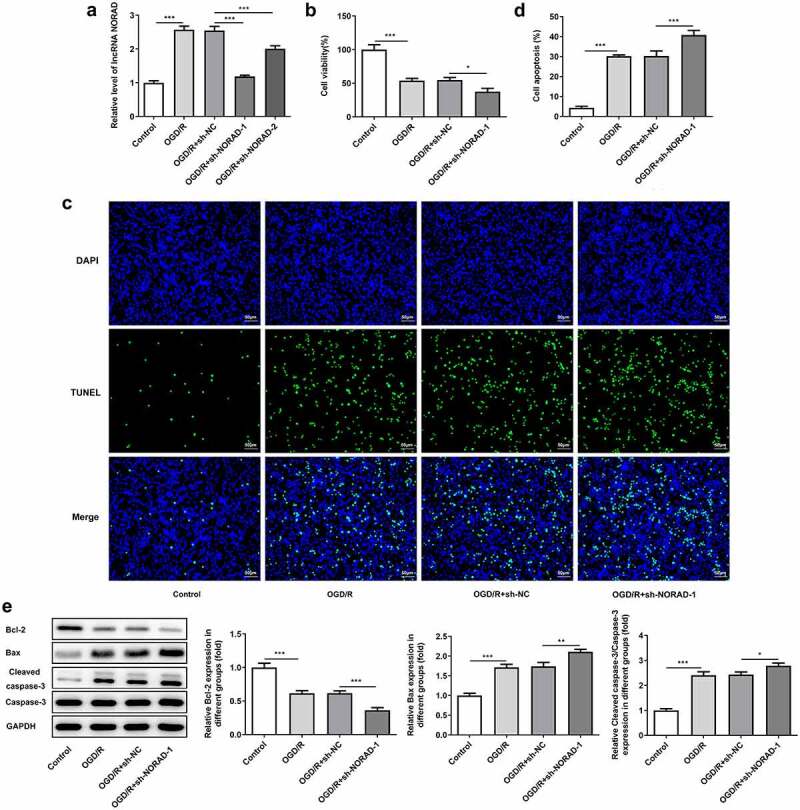
Downregulation of lncRNA NORAD reduced cell viability and promoted cell apoptosis under OGD/R condition. OGD/R-injured SH-SY5Y cells were transfected with sh-NC or sh-NORAD. (a) The silencing efficiency was validated by RT-qPCR analysis. (b) Cell viability was determined using CCK-8 assay. (c, d) Cell apoptosis was assessed using TUNEL assay. (e) Expression levels of Bcl-2, Bax and Cleaved caspase-3 were detected using western blot assay. *P < 0.05, **P < 0.01, ***P < 0.001

Moreover, SH-SY5Y cells were transfected with sh-NORAD-1 or sh-NORAD-2 and the silencing efficiency was validated by RT-qPCR analysis. Transfection with sh-NORAD-1 or sh-NORAD-2 significantly downregulated lncRNA NORAD expression and sh-NORAD-1 was chosen for the subsequent experiments due to the optimal efficiency (Figure 3(a)). CCK-8 assay indicated that downregulation of lncRNA NORAD reduced the viability of SH-SY5Y cells injured by OGD/R (Figure 3(b)). In addition, TUNEL assay and western blot analysis jointly confirmed that downregulation of lncRNA NORAD promoted the apoptosis of OGD/R-injured SH-SY5Y cells, as observed by the increased TUNEL positive cells, decreased expression of Bcl-2, as well as increased expressions of Bax and Cleaved caspase-3 (Figure 3(c-e)).

### LncRNA NORAD exerted suppressive effects on oxidative stress and inflammation under OGD/R condition

**Figure 4.**
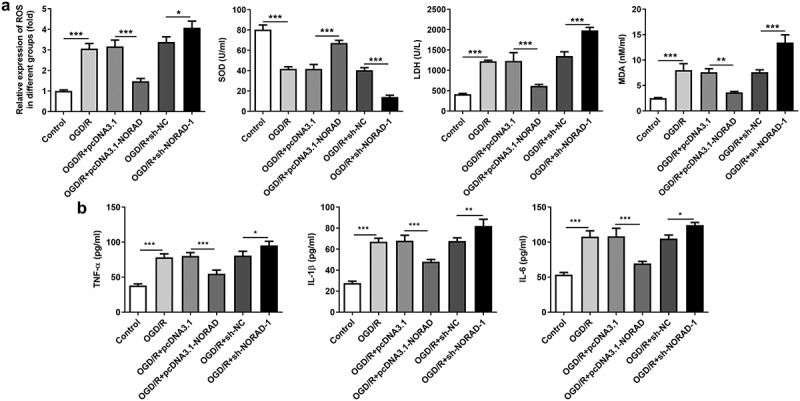
LncRNA NORAD exerted suppressive effects on oxidative stress and inflammation under OGD/R condition. OGD/R-injured SH-SY5Y cells were transfected with pcDNA3.1-NORAD or sh-NORAD. (a) The levels of ROS, MDA, LDH and SOD in cell suspension were determined by ROS Assay Kit, MDA Assay Kit, LDH Assay Kit and SOD Assay Kit. (b) The release of TNF-α, IL-1β and IL-6 was measured using ELISA Kits. *P < 0.05, **P < 0.01, ***P < 0.001

Considering the tremendous influence of oxidative stress and inflammation on cerebral ischemia/reperfusion injury, we made a deep investigation into the impacts of lncRNA NORAD on oxidative stress and inflammation in SH-SY5Y cells injured by OGD/R. Obvious oxidative stress as well as inflammation were observed upon 6 h OGD/R injury. Decreased levels of ROS, LDH and MDA and increased level of SOD following transfection with pcDNA3.1-NORAD suggested that upregulation of lncRNA NORAD alleviated oxidative stress in OGD/R-injured SH-SY5Y cells. By contrast, elevated ROS, LDH and MDA and reduced SOD induced by downregulation of lncRNA NORAD evidenced that knockdown of lncRNA NORAD aggravated oxidative stress in OGD/R-injured SH-SY5Y cells (Figure 4(a)). Meanwhile, the release of inflammatory cytokines including TNF-α, IL-1β and IL-6 was diminished upon upregulation of lncRNA NORAD and downregulation of lncRNA NORAD induced the release of inflammatory cytokines (Figure 4(b)). Collectively, these results indicated that lncRNA NORAD exerted suppressive effects on oxidative stress and inflammation in OGD/R-injured SH-SY5Y cells.

### LncRNA NORAD served as a molecular sponge for miR-30a-5p and negatively regulated miR-30a-5p expression

**Figure 5.**
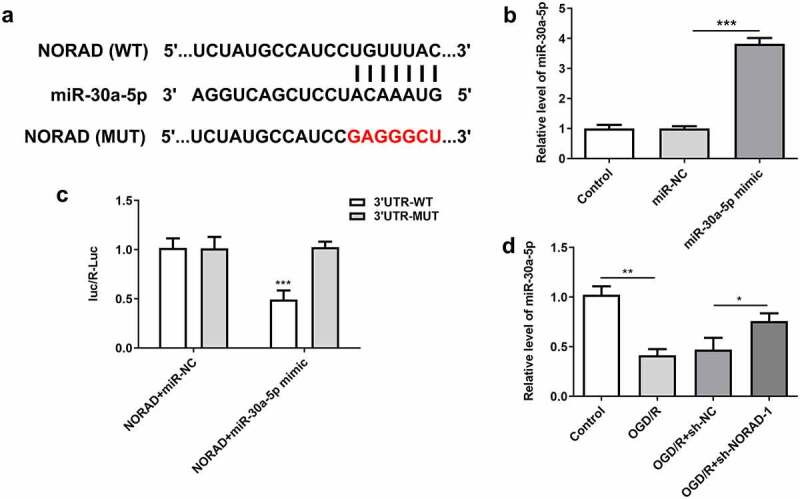
LncRNA NORAD served as a molecular sponge for miR-30a-5p and negatively regulated miR-30a-5p expression. (a) Bioinformatics analysis predicted the binding site of lncRNA NORAD to miR-30a-5p. (b) Cells were transfected with miR-30a-5p mimic and the overexpression efficiency was validated by RT-qPCR analysis. (c) Luciferase reporter assay verified the binding relationship of lncRNA NORAD and miR-30a-5p. (d) OGD/R-injured SH-SY5Y cells were transfected with sh-NC or sh-NORAD. miR-30a-5p level was detected by RT-qPCR. *P < 0.05, **P < 0.01, ***P < 0.001

To explore the molecular mechanism underlying the protective role of lncRNA NORAD in cerebral ischemia/reperfusion injury, bioinformatics analysis was employed to predict the binding relationship between lncRNA NORAD and miR-30a-5p (Figure 5(a)). Then, cells were then transfected with miR-30a-5p mimic for subsequent experiments, and the overexpression efficiency was validated by RT-qPCR analysis (Figure 5(b)). Luciferase reporter assay verified that miR-30a-5p overexpression inhibited the luciferase activity of NORAD-WT but had no obvious inhibitory effects on that of NORAD-MUT (Figure 5(c)). Additionally, downregulation of lncRNA NORAD significantly elevated miR-30a-5p expression in OGD/R-injured SH-SY5Y cells (Figure 5(d)). These data demonstrated that lncRNA NORAD sponged miR-30a-5p and negatively regulated miR-30a-5p expression.

### Upregulation of lncRNA NORAD enhanced the viability and suppressed the apoptosis of OGD/R-injured SH-SY5Y cells by repressing miR-30a-5p expression

**Figure 6.**
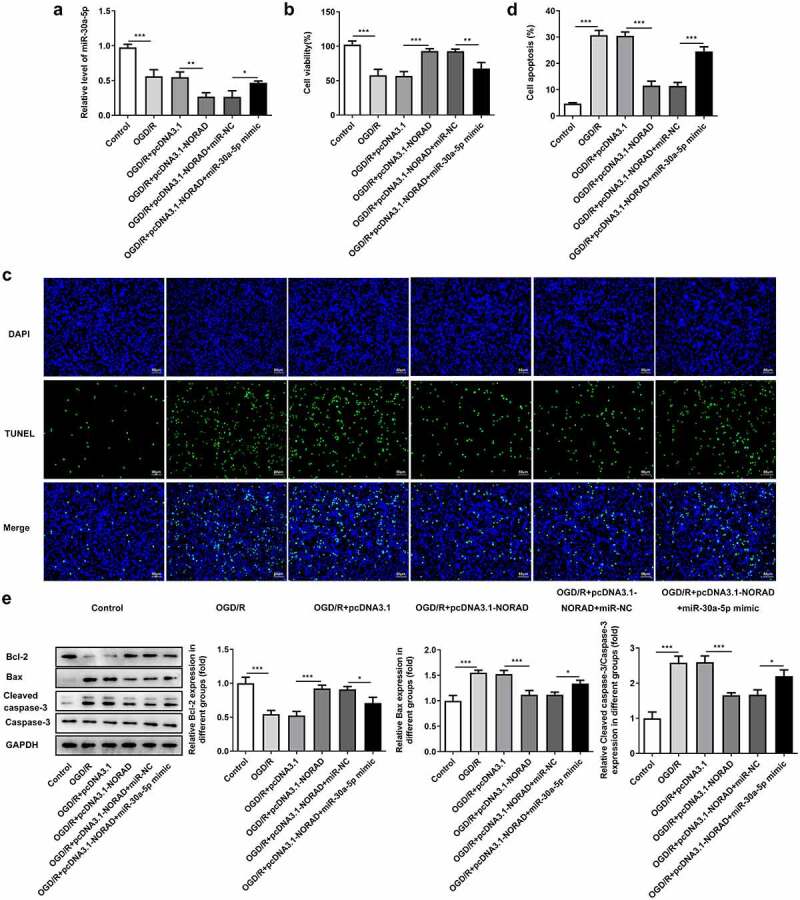
Upregulation of lncRNA NORAD enhanced the viability and suppressed the apoptosis of OGD/R-injured SH-SY5Y cells by repressing miR-30a-5p expression. OGD/R-injured SH-SY5Y cells were transfected with pcDNA3.1-NORAD or co-transfected with pcDNA3.1-NORAD and miR-30a-5p mimic. (a) miR-30a-5p level was detected by RT-qPCR. (b) Cell viability was determined using CCK-8 assay. (c, d) Cell apoptosis was assessed using TUNEL assay. (e) Expression levels of Bcl-2, Bax and Cleaved caspase-3 were detected using western blot assay. *P < 0.05, **P < 0.01, ***P < 0.001

It was observed that upregulation of lncRNA NORAD further reduced miR-30a-5p expression in OGD/R-injured SH-SY5Y cells (Figure 6(a)). The enhancement of lncRNA NORAD overexpression on the viability of OGD/R-injured SH-SY5Y cells was partially reversed after transfection with miR-30a-5p mimic (Figure 6(b)). In addition, the obviously increased TUNEL positive cells evidenced that overexpression of miR-30a-5p could exacerbate cell apoptosis, abolishing the suppressing effects of lncRNA NORAD overexpression on the apoptosis of OGD/R-injured SH-SY5Y cells (Figure 6(c, d)). Moreover, decreased expression of Bcl-2 and increased expressions of Bax and Cleaved caspase-3 induced by miR-30a-5p overexpression suggested that the inhibition of lncRNA NORAD overexpression in the apoptosis of OGD/R-injured SH-SY5Y cells was reversed by upregulation of miR-30a-5p (Figure 6(e). Overall, the upregulation of lncRNA NORAD may enhance the viability and suppress the apoptosis of OGD/R-injured SH-SY5Y cells by negatively regulating miR-30a-5p.

### Upregulation of lncRNA NORAD alleviated oxidative stress and inflammation by repressing miR-30a-5p expression

**Figure 7.**
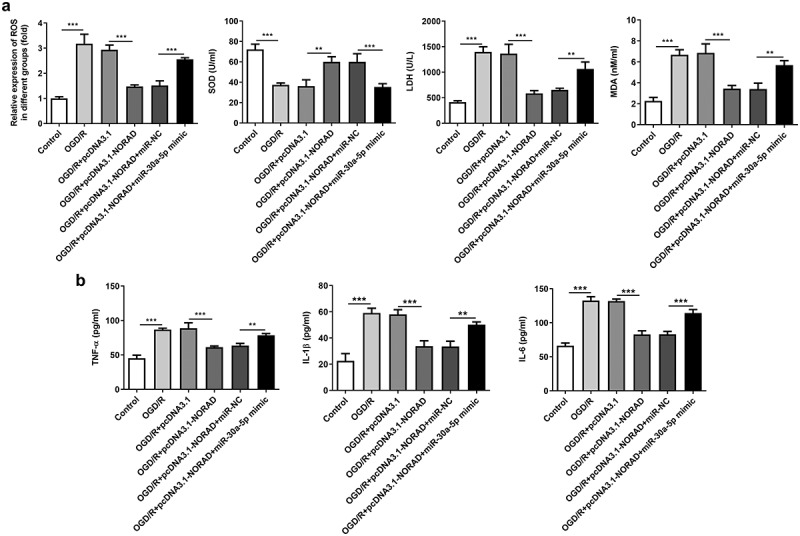
Upregulation of lncRNA NORAD alleviated oxidative stress and inflammation by repressing miR-30a-5p expression. OGD/R-injured SH-SY5Y cells were transfected with pcDNA3.1-NORAD or co-transfected with pcDNA3.1-NORAD and miR-30a-5p mimic. (a) The levels of ROS, MDA, LDH and SOD in cell suspension were determined by ROS Assay Kit, MDA Assay Kit, LDH Assay Kit and SOD Assay Kit. (b) The release of TNF-α, IL-1β and IL-6 was measured using ELISA Kits. **P < 0.01, ***P < 0.001

Then, we investigated the levels of oxidative stress markers and the release of inflammatory cytokines after co-transfection with pcDNA3.1-NORAD and miR-30a-5p mimic. An obvious decrease in SOD level and increases in ROS, LDH, MDA, TNF-α, IL-1β and IL-6 levels were observed following miR-30a-5p overexpression, suggesting that the suppressive effects of lncRNA NORAD overexpression on oxidative stress and inflammation were reversed after elevation of miR-30a-5p (Figure 7(a, b)). To sum up, upregulation of lncRNA NORAD may alleviate oxidative stress and inflammation by negatively regulating miR-30a-5p.

### LncRNA NORAD regulated YWHAG expression by sponging miR-30a-5p

**Figure 8.**
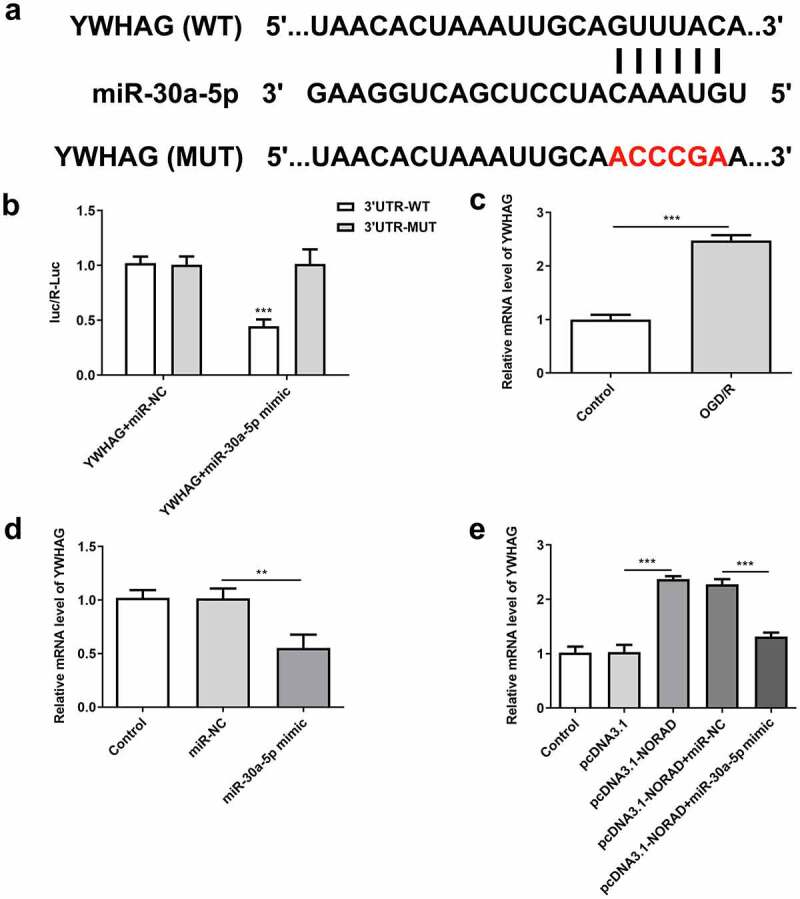
LncRNA NORAD regulated YWHAG expression by sponging miR-30a-5p. (a) Bioinformatics analysis predicted the binding site of miR-30a-5p to YWHAG. (b) Luciferase reporter assay verified the binding relationship of miR-30a-5p and YWHAG. (c) YWHAG level in OGD/R-injured SH-SY5Y cells was assessed by RT-qPCR. (d) Cells were transfected with miR-30a-5p mimic and YWHAG level was assessed by RT-qPCR. (e) Cells were transfected with pcDNA3.1-NORAD or co-transfected with pcDNA3.1-NORAD and miR-30a-5p mimic. YWHAG level was assessed by RT-qPCR. **P < 0.01, ***P < 0.001

Bioinformatics analysis also predicted the binding site of miR-30a-5p to YWHAG (Figure 8(a)). Luciferase reporter assay verified that miR-30a-5p overexpression inhibited the luciferase activity of YWHAG-WT but had no obvious inhibitory effects on that of YWHAG-MUT (Figure 8(b)). YWHAG was elevated in OGD/R-injured SH-SY5Y cells and YWHAG was notably downregulated following transfection with miR-30a-5p mimic (Figure 8(c, d)). These data demonstrated that miR-30a-5p could bind to YWHAG and negatively regulate YWHAG expression. Moreover, upregulation of lncRNA NORAD enhanced YWHAG expression, which was reversed upon elevation of miR-30a-5p (Figure 8(e)). In general, lncRNA NORAD may play a vital role in cerebral ischemia/reperfusion injury by sponging miR-30a-5p to increase YWHAG expression.

### Downregulation of lncRNA NORAD reduced the viability and promoted the apoptosis of OGD/R-injured SH-SY5Y cells by sponging miR-30a-5p to regulate YWHAG expression

**Figure 9.**
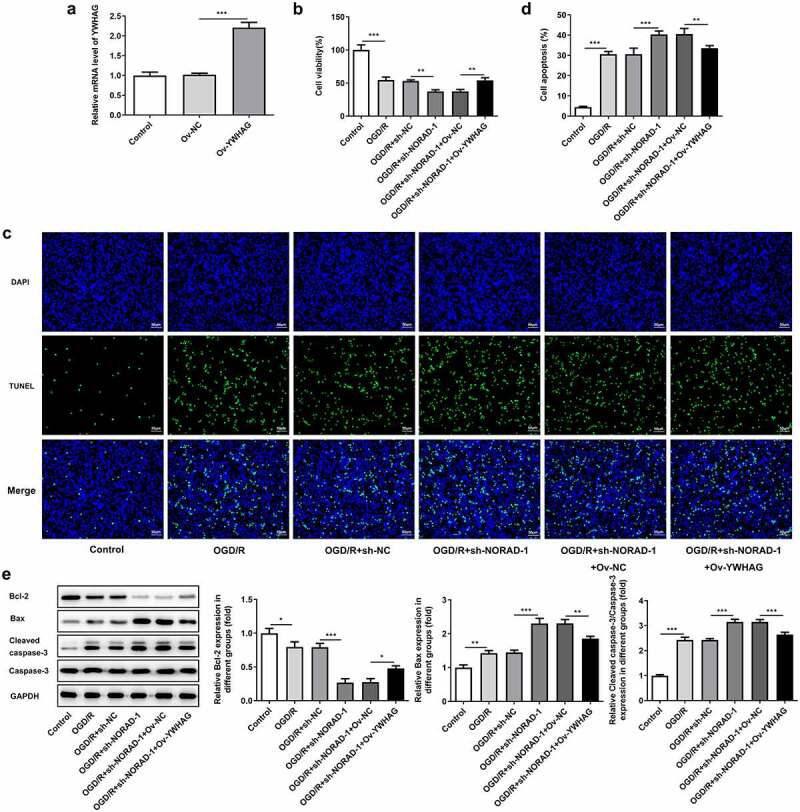
Downregulation of lncRNA NORAD reduced the viability and promoted the apoptosis of OGD/R-injured SH-SY5Y cells by sponging miR-30a-5p to regulate YWHAG expression. OGD/R-injured SH-SY5Y cells were transfected with sh-NORAD or co-transfected with sh-NORAD and Ov-YWHAG. (a) Cells were transfected with Ov-YWHAG and RT-qPCR analysis was employed to check the overexpression efficiency. (b) Cell viability was determined using CCK-8 assay. (c, d) Cell apoptosis was assessed using TUNEL assay. (e) Expression levels of Bcl-2, Bax and Cleaved caspase-3 were detected using western blot assay. *P < 0.05, **P < 0.01, ***P < 0.001

In order to determine the biological role of YWHAG in cerebral ischemia/reperfusion injury, Ov-YWHAG was introduced to upregulate YWHAG expression and RT-qPCR analysis was employed to check the overexpression efficiency (Figure 9(a)). CCK-8 assay indicated that the suppressive effects of lncRNA NORAD interference on the viability of OGD/R-injured SH-SY5Y cells were reversed following YWHAG overexpression (Figure 9(b)). Meanwhile, the obviously decreased TUNEL positive cells evidenced that elevation of YWHAG could mitigate cell apoptosis and reverse the pro-apoptotic effects of lncRNA NORAD interference in OGD/R-injured SH-SY5Y cells (Figure 9(c, d)). Additionally, western blot analysis also confirmed that YWHAG overexpression abolished the pro-apoptotic effects of lncRNA NORAD interference under OGD/R condition (Figure 9(e)). Overall, downregulation of lncRNA NORAD may reduce the viability and promote the apoptosis of OGD/R-injured SH-SY5Y cells by sponging miR-30a-5p to regulate YWHAG expression.

### Downregulation of lncRNA NORAD aggravated oxidative stress and inflammation by sponging miR-30a-5p to regulate YWHAG expression

**Figure 10.**
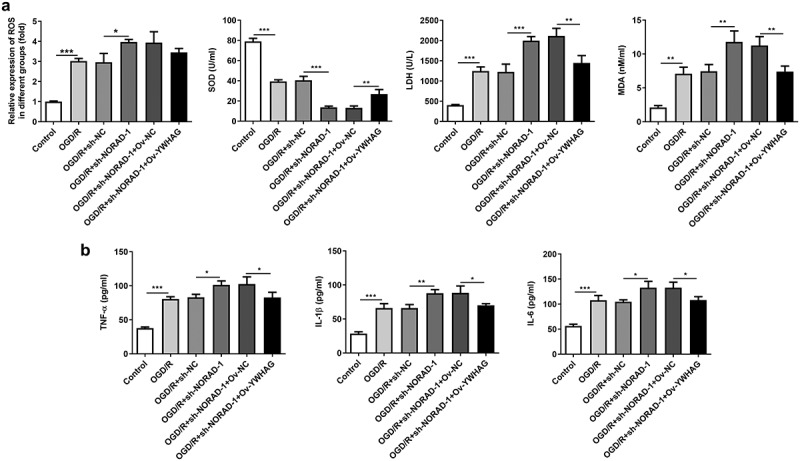
Downregulation of lncRNA NORAD aggravated oxidative stress and inflammation by sponging miR-30a-5p to regulate YWHAG expression. OGD/R-injured SH-SY5Y cells were transfected with sh-NORAD or co-transfected with sh-NORAD and Ov-YWHAG. (a) The levels of ROS, MDA, LDH and SOD in cell suspension were determined by ROS Assay Kit, MDA Assay Kit, LDH Assay Kit and SOD Assay Kit. (b) The release of TNF-α, IL-1β and IL-6 was measured using ELISA Kits. *P < 0.05, **P < 0.01, ***P < 0.001

Furthermore, the levels of oxidative stress markers and the release of inflammatory cytokines following YWHAG overexpression were determined. Increased SOD level and decreased LDH, MDA and ROS levels proved that YWHAG overexpression could significantly ease oxidative stress aggravated by downregulation of lncRNA NORAD (Figure 10(a)). Besides, the distinct decreases in TNF-α, IL-1β and IL-6 levels suggested that the promotive effects of lncRNA NORAD interference on inflammation were reversed after YWHAG overexpression (Figure 10(b)). Taken together, downregulation of lncRNA NORAD may aggravate oxidative stress and inflammation by sponging miR-30a-5p to regulate YWHAG expression.

## Discussion

With the aggravation of the aging problem, the occurrence rate of stroke has been increasing year by year, and the proportion of ischemic stroke accounts for the majority [1]. In recent years, thrombolytic therapy and mechanical thrombectomy are the major clinical treatments for ischemic stroke to open blood vessels and achieve the cerebral ischemia-reperfusion [22]. However, after recovering of blood flow from cerebral blood vessels to ischemic region, brain tissue damage is aggravated and even irreversible damage occurs [2]. Currently, the majority of neuroprotective agents have no distinct effects and the mechanism of CIRI has not been fully elucidated [5,7]. Hence, it is of great significance to look for new effective targets for treating ischemic stroke.

Recent researches have reported that lncRNAs play substantial roles in CIRI, which opens up new targets for cerebral protection [23,24]. For example, lncRNA GAS5 is upregulated in OGD/R-induced neuronal injury and inhibition of GAS5 could protect against CIRI and improve neurological functions [25]. Upregulation of lncRNA SNHG12 could alleviate CIRI by inducing autophagy activation [26]. Thus, it is of desperate need to expound the biological roles of lncRNAs in CIRI. The literature has demonstrated that lncRNA NORAD exerts anti-apoptosis effects on MPP+-induced SH-SY5Y cells [11]. In the present work, OGD/R stimulation caused a temporary increase of lncRNA NORAD levels in SH-SY5Y cells. Moreover, levels of lncRNA NORAD continuously decreased with the prolonging of stimulation time. Besides, it was demonstrated that overexpression of lncRNA NORAD enhanced the viability and suppressed the apoptosis of OGD/R-injured SH-SY5Y cells. Furthermore, the percentage of viable SH-SY5Y cells diminished upon downregulation of lncRNA NORAD. Various diseases including CIRI are complex and changeable owing to the interaction of multiple pathological links, such as oxidative stress and inflammation [5,6,27,28]. Here, we confirmed that lncRNA NORAD exerted suppressive effects on oxidative stress and inflammation in OGD/R-injured SH-SY5Y cells.

MiRNAs, widely existing in eukaryote, are a class of 18 ~ 24 nucleotides endogenous noncoding singe-stranded RNAs [29]. Numerous researches have verified that lncRNAs can interact with miRNAs to play important roles in the occurrence and development of CIRI [30,31]. Long et al. have indicated that miR-30a-5p is downregulated in patients with ischemic stroke, which could be an underlying target for the treatment of ischemic stroke [14]. Besides, downregulation of miR-30a-5p could exert the neural protective effect in ischemic stroke [15]. Herein, it was demonstrated that lncRNA NORAD served as a molecular sponge for miR-30a-5p and negatively regulated miR-30a-5p expression. Upregulation of miR-30a-5p could reduce the viability and exacerbate the apoptosis of OGD/R-injured SH-SY5Y cells, abolishing the protective effects of lncRNA NORAD overexpression against OGD/R injury. Furthermore, the suppressive effects of lncRNA NORAD overexpression on oxidative stress and inflammation in OGD/R-injured SH-SY5Y cells were reversed upon elevation of miR-30a-5p.

The 14-3-3 protein family belongs to a kind of small molecule proteins [16]. 14-3-3 proteins are regarded as ‘bridge proteins’ in protein interaction for their binding affinities to a variety of functional signal proteins [32]. As Kawamoto et al. reported, 14-3-3 proteins are reduced in astrocytes of cerebral infarction patients [18]. 14-3-3γ (YWHAG) is one of the pivotal members of the 14-3-3 protein family. Accumulating evidence has indicated that YWHAG is upregulated in OGD-stimulated primary cerebral cortical neurons, and knockdown of YWHAG promotes OGD-induced neuronal apoptosis [19]. Moreover, YWHAG could bind to p65 and regulate NF-κB signaling in rat brains after CIRI, implying that YWHAG might be an ideal therapeutic target for CIRI [20]. In this research, it was discovered that the expression of YWHAG gained an increase after OGD/R. Besides, miR-30a-5p and YWHAG could bind directly to each other, and upregulation of miR-30a-5p could effectively inhibit YWHAG expression in SH-SY5Y cells. In addition, upregulation of lncRNA NORAD enhanced YWHAG expression, which was reversed upon elevation of miR-30a-5p. LncRNA NORAD regulated YWHAG expression by sponging miR-30a-5p. Upregulation of YWHAG enhanced the viability and repressed the apoptosis of OGD/R-injured SH-SY5Y cells, abolishing the function of lncRNA NORAD knockdown in OGD/R injury. Meanwhile, the promoting effects of lncRNA NORAD knockdown on oxidative stress and inflammation in OGD/R-injured SH-SY5Y cells were reversed upon elevation of YWHAG.

## Conclusion

To conclude, we illustrated that lncRNA NORAD/miR-30a-5p/YWHAG axis may participate in the progression of CIRI. Herein, the biological functions of lncRNA NORAD/miR-30a-5p/YWHAG were investigated in OGD/R-induced neural injury in vitro. The results revealed that lncRNA NORAD exerted protective effects against OGD/R-induced neural injury, which may be attributed to the inhibition of miR-30a-5p and the enhancement of YWHAG. In addition, these findings imply that lncRNA NORAD might be a potential candidate for treating CIRI and may thus comprise appropriate therapies for ischemia/reperfusion diseases.

## Data Availability

The datasets analyzed during the current study are available from the corresponding author on reasonable request.
